# Linking social complexity and vocal complexity: a parid perspective

**DOI:** 10.1098/rstb.2011.0222

**Published:** 2012-07-05

**Authors:** Indrikis Krams, Tatjana Krama, Todd M. Freeberg, Cecilia Kullberg, Jeffrey R. Lucas

**Affiliations:** 1 Institute of Ecology and Earth Sciences, Tartu University, Tartu, Estonia; 2 Institute of Systematic Biology, Daugavpils University, Latvia; 3 Department of Psychology and Department of Ecology and Evolutionary Biology, University of Tennessee, Knoxville, TN, USA; 4 Department of Zoology, Stockholm University, Stockholm, Sweden; 5 Department of Biological Sciences, Purdue University, West Lafayette, IN, USA

**Keywords:** communication, flock, information, parid, social organization, vocal complexity

## Abstract

The Paridae family (chickadees, tits and titmice) is an interesting avian group in that species vary in important aspects of their social structure and many species have large and complex vocal repertoires. For this reason, parids represent an important set of species for testing the social complexity hypothesis for vocal communication—the notion that as groups increase in social complexity, there is a need for increased vocal complexity. Here, we describe the hypothesis and some of the early evidence that supported the hypothesis. Next, we review literature on social complexity and on vocal complexity in parids, and describe some of the studies that have made explicit tests of the social complexity hypothesis in one parid—Carolina chickadees, *Poecile carolinensis*. We conclude with a discussion, primarily from a parid perspective, of the benefits and costs of grouping and of physiological factors that might mediate the relationship between social complexity and changes in signalling behaviour.

## 1. Introduction

The social experience of an individual during ontogeny can profoundly influence vocal signals that the individual uses in its interactions with others [1,2]. Experience hearing vocal signals of, and interacting with, other individuals can shape the sounds individuals produce as well as the ways in which individuals use those sounds [3]. Recently, it has become clear that the complexity of an individual's social group can also impact the vocal signals used in its interactions with others [4]. Thus, the social group of an individual represents both a context for vocal development and a social selection pressure impacting vocal behaviour. As a result, the complexity of social groups may be a driver of the diversity and complexity of vocal signalling systems, in both a proximate (ecological) and ultimate (evolutionary) sense. In the following pages, we will address this issue of social complexity as a potential driver of vocal complexity from the standpoint of the social and vocal behaviour of a particularly complex avian family, the Paridae.

For vocal signalling systems, the social complexity hypothesis [4] argues that groups with greater social complexity will possess greater complexity in their systems of vocalizations compared with groups that are relatively simple in social structure. The social complexity hypothesis holds for either interspecific comparisons (species in which groups are socially complex compared with species in which groups are relatively simple) or intraspecific comparisons (groups that are socially complex compared with conspecific groups that are relatively simple). Social complexity can be assessed in terms of group size, group density, diversity in roles or status of group members, or diversity of relations in social networks. Vocal complexity can be measured in terms of vocal repertoire size, information (bits) in a vocal signalling system or in the diversity of ways vocal signals are used by group members. Many parid species form relatively stable flocks (in space and time), and species vary a great deal in flock size. At a basic level, then, the social complexity hypothesis would predict that species forming larger flocks should have a greater diversity of vocalizations compared with species forming smaller flocks. On the other hand, species with similar flock sizes may vary in the structure of social relations within those flocks. For example, some species are highly ‘despotic’ in that individuals in flocks form strongly linear dominance hierarchies, whereas other species are more ‘egalitarian’ in that individuals form dominance hierarchies that exhibit lower linearity owing to a greater number of dominant–subordinate reversals [5]. The greater diversity of social connections in more ‘egalitarian’ species should drive greater vocal complexity compared with the lower diversity of social connections in more ‘despotic’ species, according to the social complexity hypothesis. One of the key aims of this article is to review the work to date that speaks to these issues.

**Figure 1.**
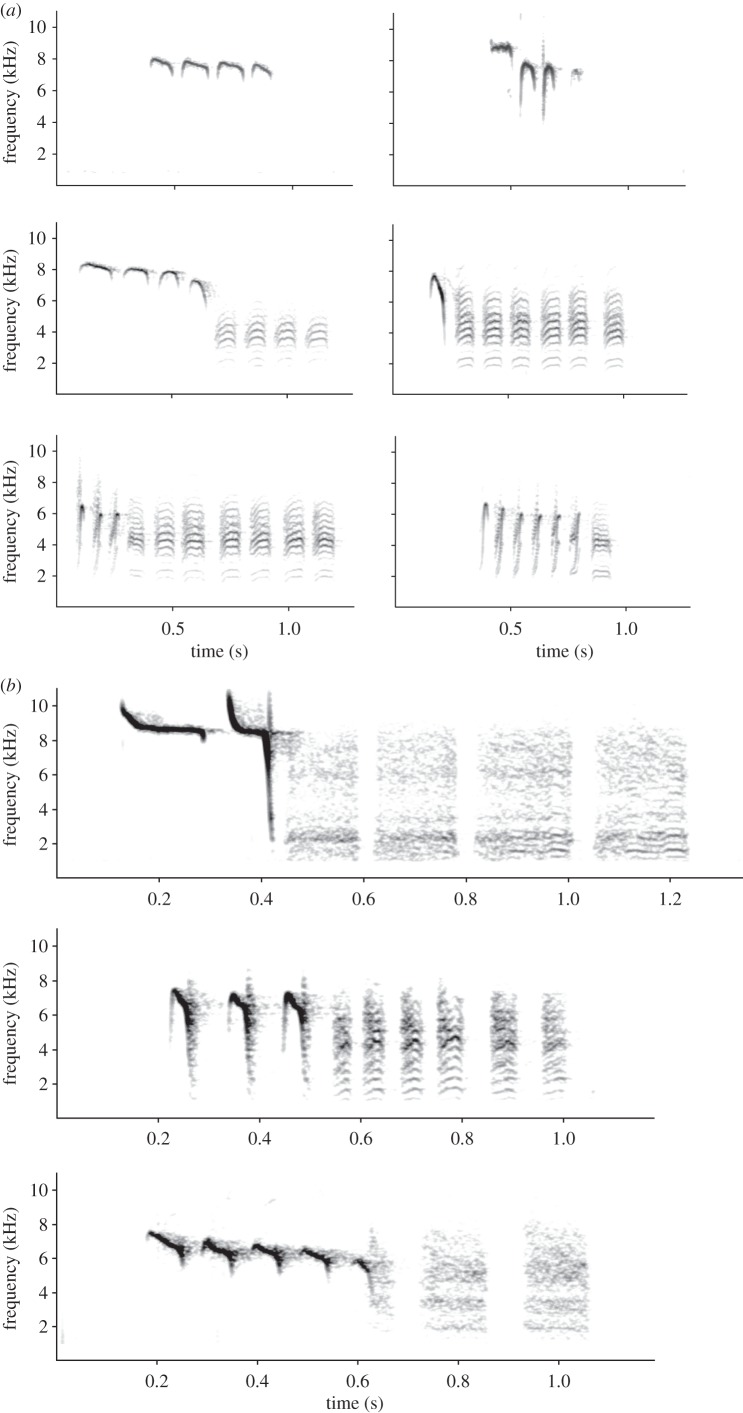
(*a*) Spectrograms of chick-a-dee calls of Carolina chickadees, *Poecile carolinensis*. (*b*) Spectrograms of chick-a-dee calls of three titmouse species: top, tufted titmouse, *Beaolophus bicolor*; middle, bridled titmouse, *B. wollweberi*; bottom, oak titmouse, *B. inornatus*. In each spectrogram, the *y*-axis represents frequency (0–11 kHz) and the *x*-axis represents time (seconds). Spectrograms were generated using Avisoft SASLab Pro (Raymund Specht, Berlin, Germany), with a fast Fourier transform (FFT) of 512, frame 100%, Blackman window function and resolution 43 Hz.

In §2, we provide a brief review of studies that have found a relationship between the social complexity of non-human mammalian and avian species and the complexity of their vocal systems. In §3, we review some of our work on this relationship in avian species of the family Paridae. This section focuses on one of the main calling systems used by members of many of these species—the ‘chick-a-dee’ call (figure 1; the ‘si-tää’ call described for willow tits, *Poecile montana*, by Haftorn [6])—and we provide an overview of the ways in which variation in the call is associated with different social, environmental and behavioural contexts. In §4, we describe some of the major benefits and costs of group living, primarily from the perspective of parid species. We conclude by linking notions of benefits and costs of social grouping (§4*c*) to questions regarding vocal signalling complexity (§4*d*), with the aim of generating ideas for future tests of the social complexity hypothesis.

## 2. Social complexity as a driver of vocal complexity

There has long been interest in relationships between the ecologies and social environments of species and variation in their systems of communication [3,7–10]. Researchers recently have tested for these relationships in a diversity of species, including visual signalling in lizards [11] and vocal signalling in sciurids [12], birds [13] and non-human primates [14]. These studies indicate that selection pressures related to living in social groups may influence species-level differences in communication.

There are many benefits of living in social groups, including an increased ability to detect and respond to predators and to detect and exploit food resources [15,16]. These abilities are often facilitated by vocalizations [7,9,17,18]. Indeed, in many species, vocal signalling aids social cohesion as group members move through their environments and may be out of visual contact with one another [1,17,19–24]. In turn, the complexity of social organization of a group likely influences the ways in which group members vocally communicate with one another. Relationships between vocal signalling and social cohesion, and between certain vocal signals and specific social interactions, have been documented in detail for many non-human primate species with complex social groups (e.g. chacma baboons, *Papio hamadryas ursinus* [25,26]; white-faced capuchins, *Cebus capucinus* [27]; golden lion tamarins, *Leontopithecus rosalia* [28]; (reviews in [17,29]).

The notion of social complexity influencing vocal complexity is also a major argument for the origin of language in humans [30,31]. Dunbar [32,33] has argued that vocal interaction in our distant *Homo sapiens* ancestors became a type of ‘grooming’ used to maintain relationships in groups—as groups became larger and larger, the number of vocal units had to increase, and how those units combined to form larger units had to become more complex, leading to the gradual emergence of an early language (Aiello & Dunbar [34] place this emergence at roughly 250 000 years ago). The question of language origin is considered one of the more difficult problems in science [35].

Comparative approaches have long provided answers to difficult problems in behaviour [36,37]. There are increasing arguments to widen our comparative approaches to questions of language origin and the evolution of complex communicative systems to include non-primate species [35,38]. Empirical work over the past two decades is starting to reveal the role social complexity might play in the evolution of complex communicative systems, and perhaps a greater understanding of this role will help us shed light on the question of the origin of language.

Comparing eight bat species, for example, Wilkinson [39] found that the information content of isolation calls of infant bats was greater for species that form larger rather than smaller colonies. Bat species that form very large colonies are thought to require greater individual-level distinctiveness in isolation calls to aid in parent–offspring recognition, which would result in greater information content at the level of the entire call system, in comparison with bats that form much smaller colonies or groups. Blumstein & Armitage [12] found that marmot and squirrel species that formed groups with a greater number of social roles had more distinct alarm calls in their vocal repertoires, relative to species that form groups with fewer social roles. In a large comparative study of non-human primates, McComb & Semple [14] found that species that form groups with greater numbers of individuals had larger vocal repertoires than did species that form groups with fewer individuals. In a study that compared social torch tail rats, *Trinomys yonenagae*, to more solitary species, de Freitas *et al.* [40] found that torch tail rats engaged in relatively higher levels of affiliative and lower levels of aggressive behaviour, and had relatively larger repertoires of vocal signals. Highly social suricates, *Suricata suricatta,* and facultatively social yellow mongooses, *Cynictis penicillata*, have larger vocal repertoires in comparison with relatively solitary mongoose species [41–43]. In each of these cases, despite the diversity of measures used by researchers, increased social complexity seems to be associated with increased vocal complexity.

Although much of the work on possible relationships between social complexity and vocal complexity has involved mammalian species, some of the work has focused on avian species. For example, many of the Psittaciformes (parrots and allies) species have quite complex social structures and have among the largest and most complex vocal repertoires, including imitation and vocal learning into adulthood [44]. The corvids (jays and crows) represent an avian family within the Passeriformes (songbirds) that may provide additional insights into relationships between social and vocal complexity [45]. For example, North American jay species differ from one another in terms of the stability or variability of their flock structure, the typical number of individuals within a flock and whether those flocks comprise unrelated or related individuals. These species, as is generally true of corvids, also have quite variable and complex systems of vocal communication. Also within Passeriformes, Kroodsma [13] found that among a group of North American wren species (family Troglodytidae), those species in which individuals faced high rates of interaction with territorial neighbours had larger repertoires of songs compared with species in which individuals faced relatively low rates of intraspecific contact and interaction.

**Figure 2.**
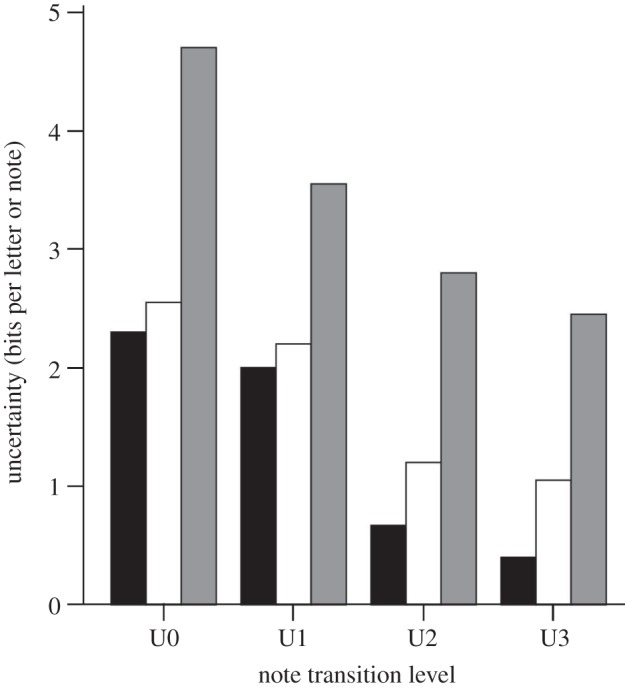
The encoding capacity (uncertainty) of the notes in chick-a-dee calls of black-capped chickadees (black bars) and Carolina chickadee (white bars) and of the letters in a sample of English text (grey bars). Figure adapted from Freeberg & Lucas [48], with data from black-capped chickadees and English words adapted from Hailman *et al.* [49]. For these different measures of complexity of note usage and note combinations, Carolina chickadee calls are qualitatively more complex than black-capped chickadee calls. This is particularly seen at higher-order levels of uncertainty that relate to how different note types are combined in pairs and triads within calls.

In the Paridae (chickadees, tits and titmice), Freeberg [46] found that Carolina chickadee, *Poecile carolinensis*, groups with many individuals had greater information content (i.e. greater uncertainty in note composition) in their chick-a-dee calls compared with groups with fewer individuals. Chick-a-dee calls are the primary social signals used in short- and medium-range communicative interactions by most parid species throughout the year [47]. In the first experimental test of whether social complexity might affect vocal complexity, Freeberg [46] manipulated group size in Carolina chickadees in captive settings, and found that birds placed into larger groups ended up using chick-a-dee calls with greater diversity of note-type usage compared with birds placed into smaller groups. More recently, Freeberg & Lucas [48] (figure 2) used several information theoretical approaches to analyse chick-a-dee calls and found that the information in calls of Carolina chickadees was qualitatively greater than for the calls of black-capped chickadees, *Poecile atricapillus* (data on black-capped chickadees came from an earlier study [49] that had compared that species’ calls to the English language in terms of structural complexity). Carolina chickadee flocks tend to be less despotic (more egalitarian—there tend to be more reversals in dominant–subordinate interactions within flocks) than black-capped chickadee flocks [50,51]. Although a comparative study of only two species limits generalizability, these results lead us to suggest that we need to focus our future efforts on understanding the network of social relations among individuals in parid flocks, if we wish to understand the social pressures that might drive communicative complexity. We now turn to specific aspects typical of parid social systems that appear to be important in the evolution of vocal complexity in this family.

## 3. Social and vocal complexity in the paridae

The Paridae family comprises three major groups of birds: the North American chickadees, the North American titmice and the Eurasian tits [52]. One reason this family is important for testing the social complexity hypothesis is that most parid species have a complex and, for songbirds, fairly atypical social organization. Broadly speaking, parid life histories tend to have the following properties [5,51,53,54]. During the spring and early summer, female and male pairs defend breeding territories and raise offspring. During late summer, young disperse from their natal grounds and the adults form flocks with other, typically non-kin, conspecifics. Flocks develop stable dominance hierarchies, and social status affects individuals’ fitness due to differential access to food and mating partners, as well as differential exposure to predators in that subordinate individuals are often forced to forage in more peripheral and exposed areas where flocks exploit food [51,55]. Flocks remain fairly stable in membership from roughly early autumn through the following spring, when they again break up into breeding pairs. Flocks are also stable in space, as members jointly defend their territory from other flocks. The complex and atypical social organization of parids has been proposed as one of the reasons for their rich vocal complexity [56,57].

Another reason parids are an important group for testing the social complexity hypothesis is that many of the species possess repertoires of diverse vocal signals. The main vocalization we address here is the chick-a-dee call (figure 1), a primary signal used to maintain group cohesion [47,49,57–59]. Chick-a-dee calls are a combinatorial vocal system in that there are a small number of distinct note types, and if a note type occurs in a given call, it can occur more than once and will follow strict note-ordering rules [49,57,60,61]. Furthermore, in black-capped chickadees, *P. atricapillus*, note types categorized by humans can be perceived as distinct note types by the birds themselves [62]. Earlier work on black-capped chickadees suggested that the chick-a-dee call was the most structurally complex vocal system known outside of human language [49,57].

**Figure 3.**
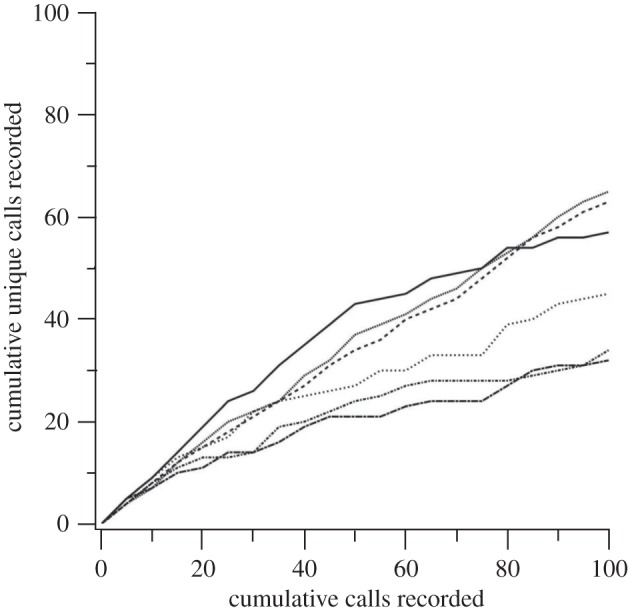
The cumulative number of novel calls in sequences of 100 calls for six Carolina chickadees continues to increase, suggesting an open-ended calling system. Each line represents a different Carolina chickadee.

One of the key reasons the chick-a-dee call is so structurally complex is that the call is an open-ended vocal system, meaning that increased recording of chick-a-dee calls will continually reveal calls with distinct note-type compositions. This open-ended nature is one of the main features the chick-a-dee call shares with human language, and one of the main differences between the chick-a-dee call and the finite song repertoires of most songbird species [63,64]. We know that the chick-a-dee call is an open-ended system based upon information theoretical analyses [65] of variation in note types across large sets of recorded chick-a-dee calls from dozens or hundreds of individuals from many different flocks [48,49]. Another way of addressing whether the call system is open-ended is to consider it from the standpoint of an individual bird. If a researcher records a large number of chick-a-dee calls from that individual, does the researcher continue to obtain unique call types as assessed by variation in note composition? If so, this continued increase in unique calls represents an open-ended system of communication. We have carried out such an analysis on sets of recordings of captive Carolina chickadees. Chick-a-dee calls of six captive Carolina chickadees that were part of the Freeberg [46] study were assessed here, as these six birds produced over 100 calls each during the study. For each bird, we considered consecutive sequences of five calls each, and determined the number of unique calls in that subsample. If the cumulative number of unique calls had quickly levelled off to an asymptote, this would have suggested that individual chickadees have a finite number of distinct calls even if call diversity at the population level fails to show an asymptote, the sort of result typically seen with repertoires of songs in songbirds. In contrast, we found that in each of the six chickadees analysed here, the cumulative number of unique calls continued to increase, with the final number of unique calls in the sample of 100 consecutive calls varying across birds (from 32 to 65 unique calls; figure 3). These data suggest either that increased recording effort would continue to find calls with a unique note-type composition, or at least that the ‘repertoire’ of chick-a-dee calls of individual Carolina chickadees is extremely large.

We can quantify the relevant properties of the cumulative distribution of call types as follows. In terms of sample coverage [66], *Θ* is the probability that an additional recorded call type will already have been documented in our sample. The sample coverage is measured as *Θ* = 1 − (*N*_1_/*I*), where *N*_1_ is the number of call types recorded only once, and *I* is the total number of calls recorded. If *Θ* is very close to unity, it means that the probability of observing a novel call type in additional recording effort is low. As *Θ* approaches or reaches unity, it suggests that the researcher has largely captured the call repertoire of an individual—the individual has reached its upper limit of call types. If *Θ* is closer to zero, however, it means that the probability of observing a novel call type with additional recording is high—that the researcher is very far from having recorded a complete vocal repertoire. In our sample of six chickadees, *I* = 100 for each and *N*_1_ ranged from 16 to 48; *Θ*s of 0.52, 0.56, 0.60, 0.74, 0.79 and 0.84. Taken together, then, the results from our cumulative unique calls which were recorded and our sample coverage analysis suggest that although chickadees do repeat call types, they continue to produce unique calls over time.

Finally, the chick-a-dee call is one of the more complex vocal systems known in non-human animals in terms of statistical information content, expressed as ‘uncertainty’ [6,49,57,60,67]. Information content is one aspect of the chick-a-dee call system that is influenced by group size manipulations [46]. Greater information here stems from considerable diversity in note compositions of calls, and an implicit assumption of ‘information’ here is that the diversity of note composition somehow maps onto distinct messages and meanings for the birds themselves. Evidence from many different laboratories and from different chickadee species indicates that the variation in chick-a-dee call structure that has been documented via information-based analyses does indeed correspond to functional variation in call structure. Increasing evidence indicates that different environmental contexts result in birds producing calls that differ in note composition, and calls differing in note composition affect receivers’ behaviour differently in playback studies (table 1); we detail some of these findings for two chickadee species below. Variation in chick-a-dee calls can affect heterospecific behaviour as well [97,98].

**Table 1.** A summary of variation in chick-a-dee call note composition or acoustic parameters of notes in relation to various identity, state or context factors.

species	factors associated with chick-a-dee call variation	references
black-capped chickadee *Poecile atricapillus*	species	[48,68–70]
	flock	[71,72]
	individual	[73]
	predator	[74–76]
boreal chickadee *P. hudsonicus*	individual	[77]
chestnut-backed chickadee *P. rufescens*	individual	[78]
Carolina chickadee *P. carolinensis*	species	[48]
	local population	[79]
	individual	[80]
	energetic state	[81]
	predator	[82–85]
	food	[87,88]
	flight	[59,82]
Mexican chickadee *P. sclateri*	individual	[89]
mountain chickadee *P. gambeli*	species	[68,69]
	individual	[90]
	flight	[91]
tufted titmouse *Baeolophus bicolor*	individual	[92]
	predator	[83,93,94]
crested tit *Lophophanes cristatus*	proximity to cover	[95,96]
willow tit *Poecile montana*	predator	[97]

Carolina chickadees provide one example of the enormous variation in the chick-a-dee call system and how that variation relates to different contexts and to individual-level variables [99]. The typical Carolina chickadee chick-a-dee call (for an eastern TN, USA, population [82]) has roughly two introductory notes (different notes types that are whistled and often show considerable frequency modulation), a C note (a noisy note type that generally increases in frequency over the course of the note) and three concluding D notes (a noisy note type with minimal frequency modulation that appears like a series of stacked overtones above a pair of carrier frequencies). Freeberg & Lucas [48] documented how the note compositional structure of the call of Carolina chickadees differs from the call of black-capped chickadees [49]. Freeberg *et al.* [79] documented local population differences in subtle acoustic features of the note types. Bloomfield *et al.* [80] demonstrated that the different note types were distinct from one another in terms of acoustic fine structure, and for each note type, it was possible to distinguish individual birds using those acoustic features. Lucas *et al.* [81] demonstrated that chickadees increase their chick-a-dee call production rate when their energy stores decline. Several laboratories have found that chickadees produce more calls and more D notes in their chick-a-dee calls when perched avian predator models have been detected [83–85]. When an avian predator is detected flying through the area where chickadees are located, those chickadees produce many fewer D notes and tend to produce more introductory notes [82]. This finding from naturalistic observational research has been corroborated with a more recent experimental study [86]. When chickadees first detect food, they produce more D notes in their calls, and this variation in their calls can affect the behaviour of receivers during playback studies [87,88]. Finally, chickadees that are in flight or on the verge of taking flight tend to produce more C notes in their chick-a-dee calls compared with when the birds are perched [59,82]. In short, for this one species, variation in this single call system can be associated with a very large number of factors and contexts related to identity, motivation and arousal related to internal and external factors, and behavioural tendencies.

Chick-a-dee calls of black-capped chickadees have also been heavily studied, and in many contexts similar to those described earlier for Carolina chickadees. For example, the note types are distinct from one another in terms of their acoustic structure, and individuals can be identified from one another based upon these acoustic differences [49,73]. The size (and therefore relative threat) and distance of predator stimuli influence the note composition of chick-a-dee calls in black-capped chickadees [74–76]. In one of the earliest studies to suggest the chick-a-dee call might function in reciprocal altruism within chickadee flocks, Ficken [101] found that black-capped chickadees produced more calls when they were in a food context, potentially recruiting flockmates to the location of the food source. In short, the findings from black-capped chickadees parallel those from Carolina chickadees, indicating that variation in the production and note composition of chick-a-dee calls is associated with a wide variety of behavioural, social and physical environmental contexts. It is still largely an open question of whether call variation is primarily externally referential or is primarily linked to emotional or arousal state, or, as we suspect, some combination of the two. Further work with chickadee species should be able to elucidate the messages and meanings of variation in calls. Increased effort to understand call variation in other chickadee, tit and titmouse species will furthermore be crucial to be able to make stronger comparative statements about the function and evolution of the call.

To summarize, many parid species exhibit an atypical and fairly complex social organization, and many appear to have a diverse and flexible calling system—the chick-a-dee call. We suggest that the complexity of social structure in parids may be one of the main reasons for the exceedingly complex call system [57]. The social pressures that stem from interacting with the same individuals over long periods of time—in agonistic and affiliative interactions, and in competitive and possibly cooperative contexts—require flexible and diverse repertoires of signals. It is incumbent upon us as researchers to understand those social pressures, however, and so in §4, we turn back to the question of parid social structure, with the aim of generating testable predictions related to our broader concern of links between social and vocal complexity.

## 4. Benefits and costs in parid social organization: future considerations for the social complexity hypothesis

In §§2 and 3, we discussed research on the question of how variation in social complexity might relate to variation in vocal complexity, with a focus on parid species. In this final section, we integrate these ideas into the broader literature on social grouping, and disease and immune system functioning in the context of sociality. We begin with a discussion of the benefits and costs of social grouping in parids and other species. We then provide a brief overview of one of the major costs of sociality—increased parasite and disease transmission—and how this cost impacts tradeoffs in immune system function. In each of these areas, we draw attention to questions that need to be addressed to gain a deeper understanding of the ways in which environmental pressures might drive changes in social structure, which in turn can impact (and perhaps be impacted by) vocal signalling complexity.

### (a) Energetic stress, predation and winter flocking

Energetic stress [102] and predation [103,104] are probably the main factors responsible for the mortality of many birds in winter. Under certain conditions, sociality can reduce the impact of both of these factors. Sociality allows savings in vigilance time without suffering increments in risk of predation, and foraging socially may reduce the risk of energetic shortfall [104,105].

All parid species studied so far are social to some extent in the non-breeding season, but the degree of sociality can vary widely between species [5]. During winter, most parids live in small coherent groups consisting of non-kin members. Prolonged juvenile association with parents is also known for the black tit, *Parus niger* [106], tufted titmouse, *Baeolophus bicolor* [107] and varied tit, *Parus varius*. Parids such as the great tit (*Parus major*), and the blue tit (*Cyanistes caeruleus*), are organized in a loose social system with no permanent group membership, and space appears not to be as rigidly partitioned into exclusive areas as in parids with discrete social units [5]. Some parid species do not form flocks at all in the overwintering months, but rather remain in the core social unit of a female–male mated pair throughout the year. For example, oak titmice (*Baeolophus inornatus*) and juniper titmice (*Baeolophus ridgwayi*) are rarely found in the winter in conspecific groups of more than two individuals [108,109]. Christman [110] argues that the handling of relatively large food items in relatively safe foraging spaces may be the evolutionary factor driving the loss of overwintering flocks in these species. If predictions of the social complexity hypothesis are supported, these two species should have relatively less complex chick-a-dee calls in comparison with tufted titmice, in which individuals form small overwintering flocks, or especially in comparison with bridled titmice, *Baeolophus wollweberi*, in which individuals can form quite large flocks [111]. This comparative question awaits formal testing, but if supported, it would beg the question of whether overwintering energetics or predation pressure is driving these changes in social organization and territoriality.

Ecological benefits of gregariousness have been addressed. For example, Ekman [112] found that willow tits, *Poecile montanus*, allocated less time per capita to scanning for predators as conspecific group size increased. Larger groups may provide an increased ability to avoid a predator, as well as a dilution effect such that an individual's chances of being attacked by a predator diminish with an increase in group size [113,114]. The very large flocks observed in bridled titmice, for example, may allow flock members to exploit better food sources that are more exposed to potential avian predators [115]. Another potential anti-predator benefit of animal aggregations is the ‘predator confusion effect’ [116] and the ‘encounter dilution’ effect [117]. Alternatively, flocking birds may often assemble around a potentially dangerous predator and move it away from the flock territory [118,119]. Many studies have shown that flocking benefits allow reallocating time to food searching (review in [15]). Increased time devoted to food searching in turn should affect fattening strategies and improve overwinter survival and fitness [120].

Behavioural interactions can show considerable plasticity depending on local biotic and abiotic conditions [121]. Especially harsh, unpredictable and physiologically stressful environments have been suggested to enhance the occurrence of positive interactions [122,123] and cooperation with neighbours [124–127] and to decrease competition [128] and delay dispersion [129,130]. Besides experimental research, there is non-experimental evidence from plant and animal communities and human societies showing a correlation between adverse conditions and cooperation [131–134]. The suite of prosocial behaviour patterns that may emerge from harsh, stressful physical environments may be part of the social pressure that demands greater communicative complexity [4]. Species in which individuals occur solitarily or in groups exhibiting strong dominance structures and little affiliative interactions would not require sets of these ‘positive’ signals.

As social complexity increases with group size (as a generality, but see [4]), each new individual potentially adds more diversity and perhaps more unpredictability to a group's communication network. In Carolina chickadees, for example, in addition to the wide range of contexts that influence chick-a-dee call structure, it appears that the ‘personality’ of an individual is also associated with its calling behaviour. Williams [135] found that more dominant/aggressive individuals tended to be bolder in contexts of novelty and increased threat, and also tended both to call more and to produce more ‘D’ notes in their calls, compared with less aggressive individuals. Similarly, Krams [95] found that dominant crested tits, *Lophophanes cristatus*, called more than non-dominant individuals. It would be important to determine in parid species whether individuals in groups with a greater diversity of ‘personalities’—that is, groups containing some balance of dominant and subordinate individuals, shy and bold individuals, highly active and less active individuals, etc.—tend to accrue greater fitness benefits than individuals in groups with more similarity among individuals [136].

### (b) Sociality, space use and mixed-species groups

Two main patterns emerge in parid associations among individuals and their use of space outside the reproductive season. The majority of temperate parids live in stable social units with a shared and exclusive area, while Eurasian great tits and blue tits have semi-stable flocks that often intermingle and live in overlapping home ranges [5]. This dichotomy in space use between stable and semi-stable flocks is tied to the use of food hoarding as an energy-management strategy. This is because hoarding is a valuable strategy only if the hoarder has relatively exclusive access to the hoarded food [5,47]. Thus, food hoarding is found primarily in species with stable territories and stable social groups. Group size and group composition may also interact with space use and hoarding behaviour.

Large numbers of conspecific individuals in flocks may increase the risk of cached food being stolen. This competitive dynamic may be one of the reasons many parids form multi-species groups consisting of a few conspecific and several heterospecific individuals [137]. Heterospecifics in such mixed-species groups appear to substitute for conspecifics as predator protection at a low competition cost [138,139]. The idea that the presence of heterospecific individuals may lessen intraspecific competition allowing more time for vigilance has been supported by studies showing that willow tits save vigilance time by associating with other tit species, goldcrests and treecreepers (see also [140]). Although interspecific competition has been observed, intraspecific competition appears to be more severe because conspecifics compete not just for food (and they may steal caches of other conspecific group members) but also for the social rank in the group's dominance hierarchy [51,141], which can be crucial for survival. On the other hand, selection for larger conspecific flocks may be driven by some unknown costs associated with flocking as a member of mixed-species groups (e.g. oddity effects seen in many fish studies (review in [15]). Large and relatively stable mixed-species flocks, in which individuals of different species respond to one anothers’ signals [98], therefore add interesting and important dimensions to the question of social complexity and the role it might play in vocal complexity.

Flock sizes of parid species vary enormously and in some species (e.g. great tits and blue tits) are hard to quantify simply because flocks are fluid and can change over time and space [5]. Nonetheless, in many species exhibiting the ‘discrete flock’ structure [5], there are very good data on flock sizes, and these sizes can differ spatially. For example, group size increases for the willow tit from two in mid-Europe, to four in Sweden, to six in Latvia, Norway and Finland as the density of congeners decreases [112,142–147]. There is also a tendency for a larger group size in North American parids compared with European parids. In general, larger conspecific groups may be interpreted as a compensation for the fewer coexisting parid species in North America, to uphold joint vigilance for predators [112].

Interspecific communication becomes an important component of the organization of mixed-species groups. For example, Magrath & Bennett [148] recently showed that superb fairy-wrens, *Malurus cyaneus*, fled to cover in response to playback of noisy miner, *Manorina melanocephala*, alarm calls associated with aerial predators. However, this response was observed only in locations where miners were present, suggesting that learning rather than acoustic structure determines the response. It therefore appears that learned eavesdropping on alarm calls may help individuals gather ecologically relevant information from heterospecifics in environments where individuals can be exposed to new species and to more complex and unpredictable social situations [99,149]. We suggest that a need for learning of the vocal repertoire of other species may be even more demanding than communicating in flocks consisting only of conspecific individuals. If this involves costly processes such as neurogenesis of the auditory or song control systems, flocking with heterospecifics may be a more expensive opportunity to replace conspecifics as predator protection than has been appreciated to date.

### (c) Costs of social grouping

Although social foraging reduces energetic costs related to food finding and vigilance, it also increases other types of costs. For example, increasing group size in wintering parids usually increases competition and leads to higher stress (especially in subordinate individuals [141,150]) and reduced resistance to parasites and pathogens. Increasing group size or group density may also increase transmission rates of parasites or disease. Very large parid flocks could potentially minimize these risks by increasing inter-individual distances and therefore decreasing group density. Bridled titmice seem to spread out more when foraging in very large groups, and an additional benefit may be that subordinate status in this species may be less costly than in other parid species [115].

In a closed social system, there is a limit to the number of social units and individuals that an area can accommodate, whereas there is no such apparent limit in open systems. Given that great tit and blue tit flocks do not defend territories or have structured dominance hierarchies, the flocks have a potential of becoming much larger than those of more territorial parids, and flocks of about 50 conspecifics have been reported in the great tit [151]. However, density-dependent variation in the risk of pathogenesis may be another generally underappreciated life-history determinant of group size in birds. For most organisms, conspecifics are the main source of disease [152]. Contact with increasing numbers of conspecifics raises the probability of infection [153] and, hence, the likelihood of needing to mount an immune response. Recently, density dependence has been tested in cooperatively breeding birds, which are expected to suffer from higher costs of parasitism than pair-breeding species. Sociality should facilitate horizontal transmission and possibly select for higher parasite virulence [154]. It was predicted that cooperative breeders should invest relatively more in immune defence than closely related species that breed in pairs [155,156]. Spottiswoode [157] showed that the response of the immune system to the mitogenic lectin, phytohaemagglutinin (response), was significantly higher in cooperative breeders, suggesting additional costs of philopatry and helping behaviour might be imposed on social individuals in larger groups. Increased investment in the immune system may also be important for birds that spend their winter season as members of social groups. If so, studies should reveal important associations between immune system response and various metrics of social complexity.

### (d) Parid social and vocal complexity

We have emphasized work on parids and the chick-a-dee call system here. Over two decades ago, Hailman *et al*. [49] showed that the chick-a-dee call system was one of the most complex call systems ever described in terms of its open-ended nature and the potential information conveyed by the system. Our review underscores the fact that this call system is an ideal model system for testing the social complexity hypothesis and for understanding the causal mechanisms driving the relationship between social complexity and vocal complexity. Although the chick-a-dee call has been well studied over the past few decades, there is still a great deal we do not know about its development, function and evolution. We have some exciting evidence in at least one species—Carolina chickadees—that experimental changes in social complexity can drive changes in complexity of the chick-a-dee call. More comparative data from a wider range of species are greatly needed. For example, we know very little about the vocal behaviour and social structure of African parids in the species-rich Melaniparus group.

Social behaviour, space use, energy and cache regulation, and socially induced costs imposed by disease and parasite pressure are all traits that impact group size and group cohesion. The complexity and stability of social ties, in addition to group size, in turn appear to be evolutionary forces that drive vocal complexity. There is even some evidence that seasonal changes in social complexity result in variation in the use of the chick-a-dee call [158]. We have focused on the chick-a-dee call system here, but it is important to stress that this call system is but one of the sets of vocal signals produced by parids. Like other songbird species, parids produce songs (typically males, during the breeding season) [56]. Recent work on another vocal system in parids suggests important links to the question of social complexity. The ‘gargle’ system is used by parids primarily in the context of agonistic interactions at a close range [159]. The social complexity hypothesis would predict that individuals in more socially complex groups would produce a greater diversity of vocal signals compared with individuals in less socially complex groups. Turning back to the North American titmice species discussed earlier, we would predict individual bridled titmice, which often form quite large flocks, to use a wider variety of vocal signals in comparison with individual juniper titmice, which are rarely found in conspecific groups larger than two individuals.

We have reviewed much of what is known about vocal complexity in parids, and have made links to different aspects of sociality in these species. Our bigger aim with this article, however, was to raise a number of questions to generate research ideas. This is a fascinating and, we think, important family with which to answer questions about relationships between social complexity and vocal complexity. There is considerable variation across species in fundamental social dimensions such as group size, presence and number of heterospecifics in mixed-species flocks, presence or absence of territories, and therefore social network metrics. There also appears to be considerable variation in the structure and use of chick-a-dee calls, let alone the broader repertoires of different vocal signals used by each species. Social and vocal behavioural data are needed for a greater number of parids if we are to be able to do the fundamental comparative work [160] that will help us determine the evolution of vocal complexity in these species.
